# Regulation of melanosome number, shape and movement in the zebrafish retinal pigment epithelium by OA1 and PMEL

**DOI:** 10.1242/jcs.164400

**Published:** 2015-04-01

**Authors:** Thomas Burgoyne, Marie N. O'Connor, Miguel C. Seabra, Daniel F. Cutler, Clare E. Futter

**Affiliations:** 1 UCL Institute of Ophthalmology, London EC1V 9EL, UK; 2 MRC Laboratory for Molecular Cell Biology, University College, London WC1E 6BT, UK; 3 NHLI, Imperial College, London SW7 2AZ, UK

**Keywords:** OA1, PMEL, Melanosome, Retinal pigment epithelium

## Abstract

Analysis of melanosome biogenesis in the retinal pigment epithelium (RPE) is challenging because it occurs predominantly in a short embryonic time window. Here, we show that the zebrafish provides an ideal model system for studying this process because in the RPE the timing of melanosome biogenesis facilitates molecular manipulation using morpholinos. Morpholino-mediated knockdown of OA1 (also known as GPR143), mutations in the human homologue of which cause the most common form of human ocular albinism, induces a major reduction in melanosome number, recapitulating a key feature of the mammalian disease where reduced melanosome numbers precede macromelanosome formation. We further show that PMEL, a key component of mammalian melanosome biogenesis, is required for the generation of cylindrical melanosomes in zebrafish, which in turn is required for melanosome movement into the apical processes and maintenance of photoreceptor integrity. Spherical and cylindrical melanosomes containing similar melanin volumes co-exist in the cell body but only cylindrical melanosomes enter the apical processes. Taken together, our findings indicate that melanosome number and shape are independently regulated and that melanosome shape controls a function in the RPE that depends on localisation in the apical processes.

## INTRODUCTION

The retinal pigment epithelium (RPE) lies between the outer segments of the photoreceptors and the choriocapillaris, a layer of fenestrated capillaries bringing blood to the retina. The RPE maintains the photoreceptors, providing nutrients and removing waste products, phagocytosing shed outer segments and regenerating photopigment in the visual cycle (Strauss, 2005). Additionally, the RPE is densely packed with melanosomes containing melanin pigment, which can reduce harmful backscattered light and remove free radicals that arise during phagocytosis of photoreceptor outer segments (Strauss, 2005). Defects in melanosome biogenesis and movement are associated with human retinal disease, and a deficit of melanin pigment in the RPE is associated with aging and age-related macular degeneration. Although the potential protective effects of melanin are well established, the precise roles of melanosomes and their movement in the RPE and the molecular regulation of their biogenesis and movement remains to be fully established.

The most common form of ocular albinism is caused by mutation of the heterotrimeric G protein coupled receptor, OA1 (also known as GPR143) (Bassi et al., 1995; d'Addio et al., 2000). This disease is characterised by a reduced number of enlarged melanosomes and is associated with visual defects. In the OA1-knockout mouse, melanosome number is reduced early in development before the increased melanosome size (Incerti et al., 2000), suggesting that melanosome number and size are independently regulated. OA1 also regulates microtubule-dependent melanosome movement (Palmisano et al., 2008) and its multiplicity of roles has made the role of OA1 in individual trafficking steps in the RPE difficult to dissect. Also hampering the elucidation of the function of OA1 and other melanosome regulators in the RPE is the short time window for melanosome biogenesis in embryonic life and the scarcity of RPE cell culture systems that make melanosomes. For this reason, the majority of studies on melanosome biogenesis have been performed in melanocytes, where four stages of maturation (I–IV) have been described (Seiji et al., 1963). From studies on cultured melanocytes, melanosomes have been shown to derive from multivesicular endosomes/bodies (MVBs) (Raposo et al., 2001). Within those MVBs (stage I melanosomes), a pigmented-cell-specific protein, PMEL, undergoes proteolytic processing, leading to the generation of fibrils upon which melanin is deposited (Berson et al., 2001). Proteolytic fragments of PMEL undergo CD63-dependent sorting onto intraluminal vesicles (ILVs) where PMEL is processed and polymerises, forming an amyloid fibril matrix (van Niel et al., 2011; Watt et al., 2013). PMEL-dependent fibril formation has previously been shown to be required for melanosome elongation during mammalian melanosome biogenesis in melanocytes (Hellström et al., 2011; Theos et al., 2006). As the fibrils expand and extend across the length of the melanosome, this process promotes elongation of the immature melanosome (Hurbain et al., 2008). Melanin-synthesising enzymes delivered to the immature (stage II) melanosome catalyse the deposition of melanin upon the fibrils to form stage III melanosomes (Berson et al., 2001; Raposo et al., 2001). During subsequent maturation to stage IV, in which fibrils are no longer visible, the melanocytes gains the capacity to transfer melanosomes to neighbouring keratinocytes, in a process requiring a Rab27a-dependent interaction with the actin cytoskeleton (Hume et al., 2001; Wu et al., 2001; Wu et al., 2002). Although a similar process of melanosome biogenesis appears to occur in mammalian RPE (Futter et al., 2004), the mature melanosomes are retained in the largely postmitotic RPE cells throughout life. RPE melanosomes do, however, undergo Rab27a-dependent interaction with the actin cytoskeleton (Futter et al., 2004; Gibbs et al., 2004) that allows access to the apical processes that are in intimate contact with the photoreceptor outer segments. In fish and amphibians, this light-dependent movement along the apical processes is much more extensive than in mammals, providing opportunities for analyses of this aspect of their behaviour (Burnside and Laties, 1979).

In this study, we assess the zebrafish as a potential model in which to determine the molecular regulation of melanosome biogenesis and movement in the RPE and the role of defects in these processes in human retinal disease. We focus on two key regulators of melanosome biogenesis in mammalian cells, OA1 and PMEL, to demonstrate the potential utility of the zebrafish as a model system and reveal a novel relationship between melanosome biogenesis and movement.

## RESULTS

### OA1 morpholinos reduce melanosome number at 2 dpf without causing increased melanosome size

**Fig. 1.**
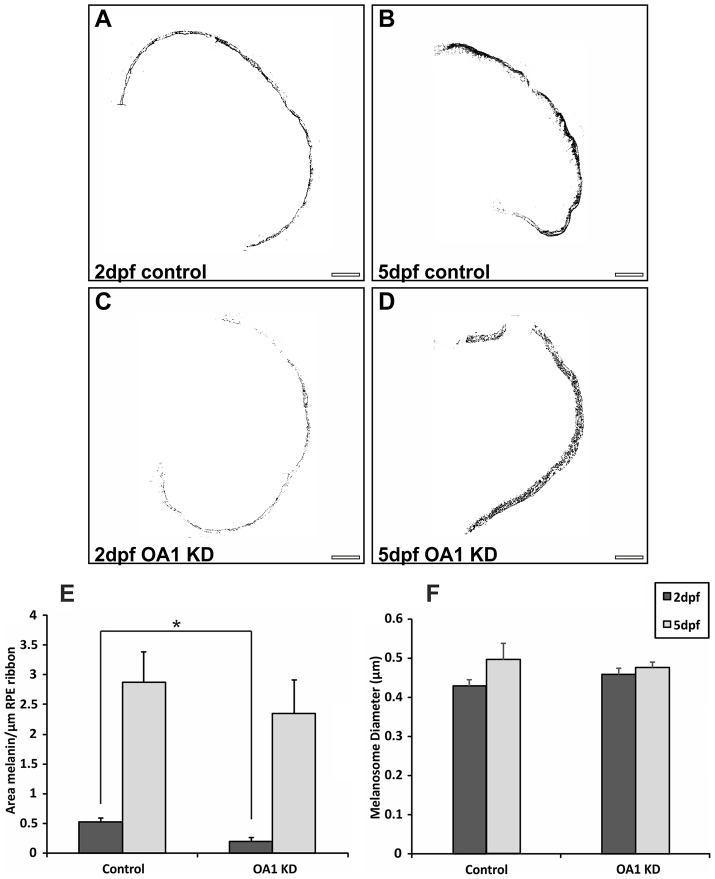
**OA1 MOs reduce melanosome number at 2 dpf without affecting melanosome size.** (A–D) Contrast-enhanced images of zebrafish eye cross-sections highlighting only electron-dense melanin. Images are shown from (A,B) control and (C,D) OA1 MO-treated zebrafish. KD, knockdown. Scale bars: 20 µm. (E) At 2 dpf there is a significant reduction in melanin area between controls and OA1 MO-treated zebrafish. By 5 dpf, the OA1 MO appears to be less effective, resulting in no significant difference in melanin area between controls and OA1 MO-treated animals. (F) The OA1 MO had no apparent effect on melanosome diameter at 2 and 5 dpf. Results show the mean±s.e.m.; *P<0.05 (Student's *t*-test).

To compare the regulation of melanosome biogenesis in zebrafish with that in mammalian cells and to evaluate zebrafish as a model to study human disease, the transcript for zebrafish OA1 was targeted with antisense morpholinos (MOs). At 2 and 5 days post-fertilisation (dpf) larvae were fixed and processed for transmission electron microscopy (TEM). It was immediately apparent from ultrathin sections through the entire retina of zebrafish embryos that there is a huge increase in melanin production between 2 and 5 dpf. Image thresholding allowed the area of melanin in the RPE at 2 and 5 dpf to be quantified. Correcting for a small increase in melanosome size between these two ages, the area of melanin indicates that there is a large (greater than fivefold) increase in melanosome number between 2 and 5 dpf (Fig. 1). Embryos depleted of OA1 could be identified by their reduced pigmentation at 2 dpf but the difference between OA1 targeting and control-morpholino-treated embryos was less clear at 5 dpf (supplementary material Fig. S1). TEM analysis of OA1 MO-treated zebrafish larvae showed no difference in melanosome size or distribution compared with that of controls, but there was a significant reduction in melanosome area and, therefore, number at 2 dpf (Fig. 1). This corroborates mammalian studies where OA1-knockout mice have been found to show reduced melanosome numbers at birth prior to a latent defect in melanosome size and distribution. By 5 dpf, melanosome numbers had largely recovered in larvae treated with OA1 MOs, presumably owing to the loss of MO efficacy due to dilution as the larvae increase in size.

### Intense melanosome production occurs in the RPE at 2 dpf but most melanosomes appear to be mature

**Fig. 2.**
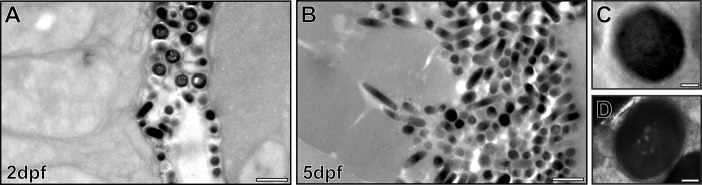
**Intense biogenesis of mature melanosomes between 2 and 5 dpf.** Electron micrographs of zebrafish RPE show a large increase in melanosome number between 2 dpf (A) and 5 dpf (B). (C,D) High-magnification images of mature melanosomes at 2 dpf. Some mature melanosomes have an appearance of ‘holes’ (D). Scale bars: 1 µm (A,B), 100 nm (C,D).

In spite of this intense melanosome biogenesis, the vast majority of melanosomes at 2 dpf were densely packed with melanin and therefore appeared to be mature stage IV melanosomes (Fig. 2). We were unable to identify immature stage II melanosomes (containing visible fibrils without melanin) and stage III melanosomes (containing fibrils upon which melanin has been deposited). The mature melanosomes sometimes appeared to contain ‘holes’, suggestive of the lumen of ILVs (Fig. 2; supplementary material Fig. S2) in zebrafish RPE. However, we could find very similar profiles in mammalian RPE cells (supplementary material Fig. S2).

### Tyrosinase morpholinos reveal potential immature melanosomes in the RPE

The absence of morphologically identifiable immature melanosomes at 2 dpf suggested that melanosome maturation might be so rapid that immature melanosomes are very short lived. We have previously found in mammalian RPE that inhibition of melanosome maturation by functional deletion of tyrosinase, the rate-limiting enzyme for melanin synthesis, transiently causes a major increase in immature melanosome numbers, as they are unable to mature beyond stage II (Lopes et al., 2007b). To simulate a similar model in zebrafish, tyrosinase MOs were used to inhibit melanosome maturation.

**Fig. 3.**
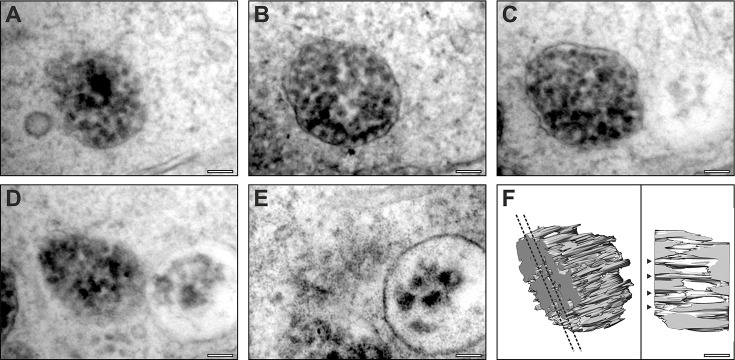
**Serial section electron microscopy analysis of tyrosinase MO-treated RPE reveals immature melanosomes that contain fibrils.** (A–E) Serial section micrographs of the same immature melanosome in an RPE cell from a tyrosinase MO-treated zebrafish at 5 dpf. (F) 3D rendering of the micrograph data reveals fibrils running through the melanosome. Dotted lines indicate the position of the slice shown in the right-hand panel; arrowheads indicate fibrils running through the entire thickness of the melanosome. Scale bars: 100 nm.

Successful MO-mediated tyrosinase depletion was readily identified through diluted skin colour of the embryos (supplementary material Fig. S1) but, at 2 dpf, fibrillar stage II melanosomes could still not be readily identified in the RPE. However, by 5 dpf, when the MO was becoming less effective, the presence of small amounts of melanin made immature melanosomes easy to identify. Within these immature melanosomes, melanin appeared to be deposited as dense spots within the MVB structures, rather than on fibrils (Fig. 3; supplementary material Fig. S3). Initially it was difficult to discern whether the spots were ILVs surrounded by melanin or PMEL fibrils running into the depth of the section. Utilising serial section electron microscopy, the dense melanin spots were revealed as fibrils running perpendicular to the plane of view (Fig. 3; supplementary material Fig. S3), suggesting that, as has been described in mammalian RPE, melanin is deposited on fibrils in immature melanosomes. Indeed, on the rare occasions when an immature melanosome was sectioned parallel to the longitudinal axis, fibrils on which melanin had been deposited could be discerned, and so these resembled stage III melanosomes (supplementary material Fig. S3). It is noteworthy that melanin in immature fibrillar melanosomes in mouse RPE appears as dense spots in melanosomes sectioned perpendicular to the fibrils (supplementary material Fig. S2).

### Two populations of melanosomes co-exist in the RPE cell body but only cylindrical ones enter the apical processes

**Fig. 4.**
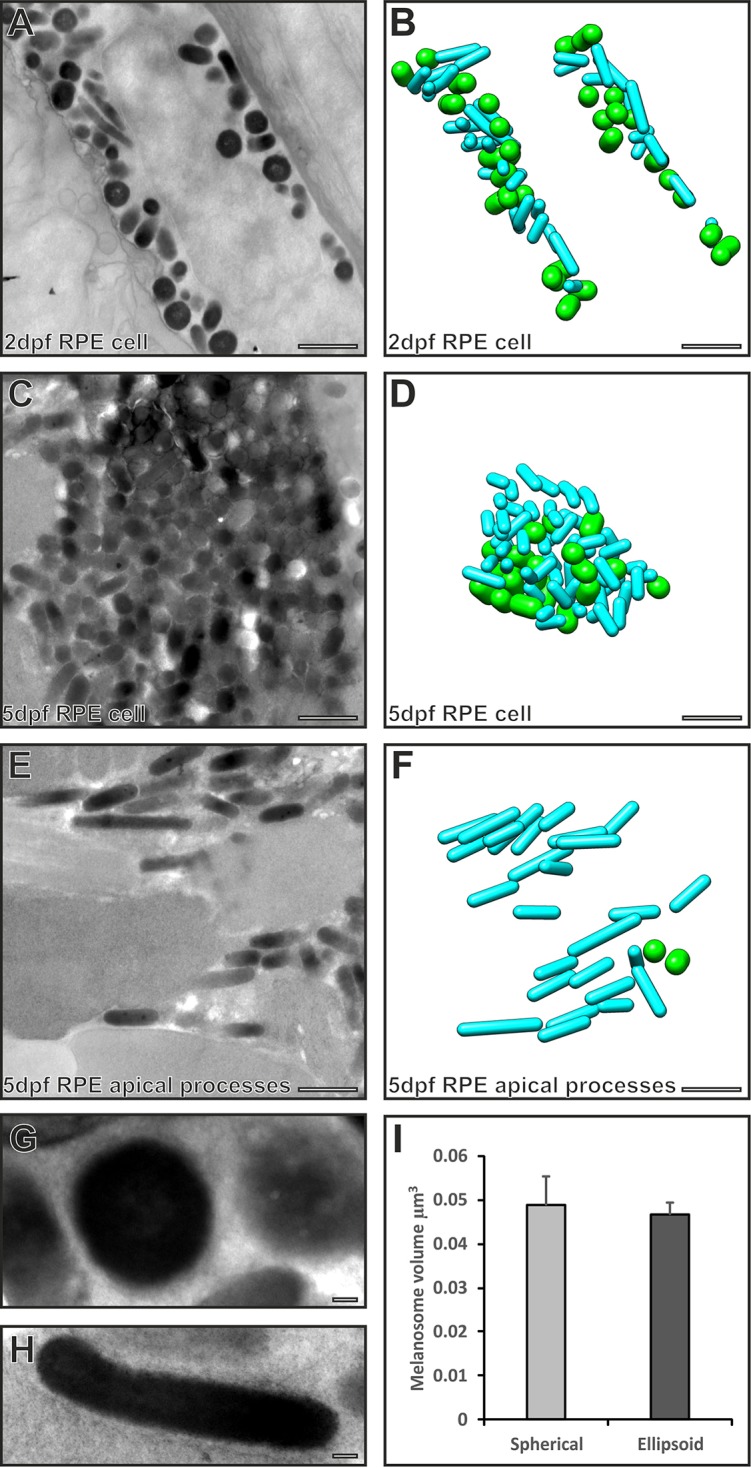
**Serial section electron microscopy analysis reveals two populations of melanosomes in the RPE cell body, but only cylindrical ones enter the apical processes.** (A,C,E) Single electron micrographs from a stack of serial section images used to generate models (B,D,F) of spherical (green) and cylindrical (cyan) melanosomes. (A,B) Both cylindrical and spherical melanosomes are found in the RPE cell body at 2 dpf. (C,D) Cylindrical and spherical melanosomes are densely packed in the RPE cell body at 5 dpf. (E,F) Only cylindrical melanosomes are in the apical processes at 5 dpf. (G,H) Higher-magnification images of a spherical melanosome (G) and a cylindrical melanosome (H). Scale bars: 1 µm (A–F), 100 nm (G–H). (I) Spherical and cylindrical melanosomes have similar volumes. Results show the mean±s.e.m.

Fibril elongation within immature melanosomes has previously been shown to promote melanosome elongation. Melanosomes are very densely packed in zebrafish RPE compared with mammalian cells, and the majority of melanosomes at 2 dpf appeared to be spherical (Fig. 2). Owing to the tight arrangement of melanosomes, it is conceivable that most were oriented with the long axis parallel with the basal membrane. If this were the case, it could infer an inaccurate interpretation of the melanosome shape in thin sections. To elucidate the melanosome shape and orientation in three dimensions, serial TEM sections were examined. This revealed two distinct types of melanosome, larger spherical and thinner elongated melanosomes (Fig. 4). Although previously described as ‘elliptical’, three dimensional (3D) analysis showed that the elongated melanosomes were, in fact, cylindrical (with a constant core diameter) with rounded ends. Both types were present at 2 and 5 dpf and their 3D orientation appeared arbitrary in the cell body. Apical processes were absent at 2 dpf but were present at 5 dpf and contained exclusively cylindrical melanosomes aligned parallel alongside photoreceptor outer segments. Cylindrical and spherical melanosomes had very similar volumes, indicating that they contained similar amounts of melanin. The diameter of cylindrical melanosomes was less than that of spherical melanosomes, and cylindrical melanosomes had a higher surface∶volume ratio.

### PMELa MOs inhibit the formation of cylindrical melanosomes

To determine whether the generation of cylindrical melanosomes requires PMEL in a manner reflecting that of mammalian RPE melanosomes, the effect of PMEL MOs on melanosome shape was analysed. Unlike human and mouse RPE, zebrafish express two forms of PMEL, termed PMELa and PMELb, (Schonthaler et al., 2005). PMELb has shorter polycystic kidney disease (PKD) and RPT (repeat domain) regions than PMELa (supplementary material Fig. S4A). Deletion studies in mammalian cells have implicated the RPT region in the appearance of fibrils and in their periodicity (Leonhardt et al., 2013).

**Fig. 5.**
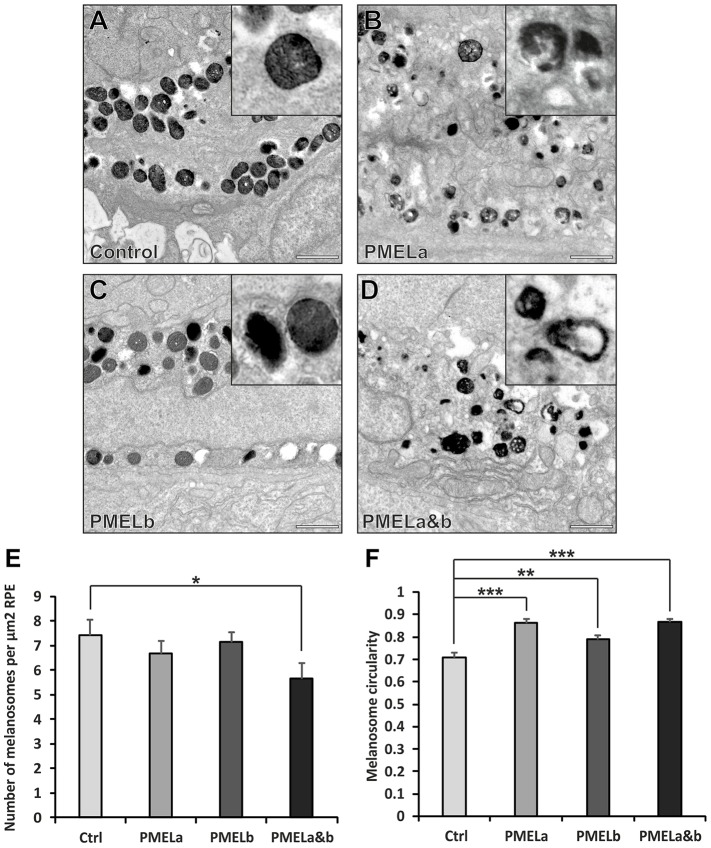
**PMELa MO reduces the number of cylindrical melanosomes to a greater extent than PMELb MO.** (A–D) Electron micrographs highlighting melanosome morphology at 2 dpf for zebrafish injected with control (A), PMELa (B), PMELb (C) and PMELa&b MOs (D). Scale bars: 1 µm. (E) The PMELa&b MO resulted in a decrease in the number of melanosomes. Ctrl, control. (F) All PMEL MOs resulted in a significant loss of cylindrical melanosomes. PMELa had a greater effect on melanosome shape than the PMELb MO. Results show the mean±s.e.m. *P<0.05, **P<0.01, ***P<0.001 (Student's *t*-test).

PMELa and PMELa&b MOs caused a clear disruption of melanosome morphology at 2 dpf (Fig. 5). There was a small decrease in melanosome number after knockdown of PMELa and PMELa&b (Fig. 5). Measuring the circularity of the melanosomes revealed that there was a preferential loss of cylindrical melanosomes in the PMELa and PMELa&b MO-treated larvae, which was less evident in the PMELb MO-treated larvae (Fig. 5; supplementary material Fig. S4B). This implies that PMELa in particular is required for the generation of cylindrical melanosomes in zebrafish.

### PMELa morpholinos prohibit melanosome movement into the apical processes and affect photoreceptor morphology

**Fig. 6.**
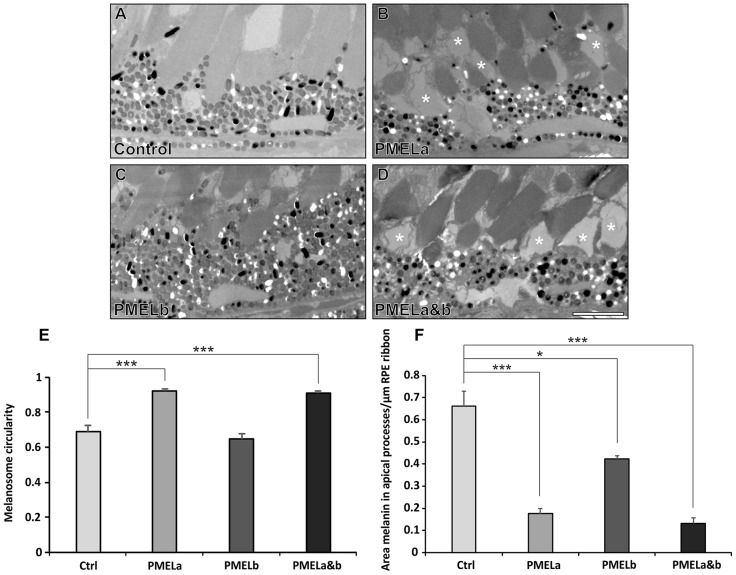
**PMELa MO prevents melanosome movement into the apical processes and has a profound effect on photoreceptor morphology.** (A–D) Electron micrographs showing the effect on melanosome positioning in the apical processes at 5 dpf for zebrafish injected with control (A), PMELa (B), PMELb (C) and PMELa&b MOs (D). (B,D) PMELa and PMELa&b MOs resulted in missing or disrupted outer segments as shown by the asterisks. Scale bars: 4 µm. (E) Photoreceptors in fish injected with PMELa and PMELa&b MOs had significantly fewer cylindrical melanosomes at 5 dpf. Ctrl, control. (F) PMELa and PMELa&b MOs resulted in a reduced area of melanin (thus fewer melanosomes) in the apical processes. Results show the mean±s.e.m.; *P<0.05, ***P<0.001 (Student's *t*-test).

Similar to the results at 2 dpf, at 5 dpf, RPE melanosomes in PMELa and PMELa&b MO-injected larvae were mostly spherical (Fig. 6; supplementary material Fig. 4C) but, in addition, there was significant loss of melanosomes in the apical processes (Fig. 6). Therefore, it appeared that the larger spherical melanosomes were not able to gain access to the apical processes, demonstrating a clear link between melanosome shape and position within RPE cells that is dependent upon PMELa expression.

At 2 dpf, photoreceptor outer segments have not yet formed but, at 5 dpf, immature photoreceptor outer segments are present. PMELa and PMELa&b MOs clearly affected photoreceptor outer segment integrity, with lost or damaged outer segments observed. Consistent with a less-severe melanosome shape and movement phenotype, photoreceptor outer segments were also less affected in the PMELb MO-injected zebrafish.

## DISCUSSION

The short time window for RPE melanosome biogenesis renders this a difficult process to study in mammalian models (Lopes et al., 2007b). The huge increase in melanosome number in zebrafish RPE between 2 and 5 dpf, when injected MOs are effective, suggests zebrafish as a model organism for the study of molecular mechanisms of melanosome biogenesis in the RPE. Furthermore, using OA1-targeted MOs to simulate the most common cause of ocular albinism recapitulates the reduced melanosome numbers observed in the human disease. Melanosome size was not affected following treatment with OA1 MOs, similar to results obtained with the OA1 knockout mouse, in which melanosome numbers are reduced at birth but increased melanosome size only develops at a later stage. Our data thus support the independent regulation of melanosome size and number by OA1 activity.

Despite the intense melanosome biogenesis between 2 and 5 dpf, fibrillar immature melanosomes were difficult to identify. The apparent absence of fibrillar immature melanosomes, together with the presence of ‘holes’ in mature melanosomes, has previously led to the proposal that, in zebrafish, melanin is deposited around the ILVs of MVBs, rather than upon fibrils (Turner et al., 1975). In mouse RPE, the identification of early fibrillar (stage II) melanosomes is facilitated by loss of function of tyrosinase, which prevents melanin synthesis (Lopes et al., 2007b). A similar approach in zebrafish, using tyrosinase MOs, also facilitated the identification of immature melanosomes, which had a speckled appearance that resembled melanin on ILVs as described previously in goldfish (Turner et al., 1975). However, here, serial section analysis reveals that these speckles of melanin are, in fact, melanin-coated fibrils running perpendicular to the plane of view. It appears, therefore, that in the zebrafish RPE fibrils within immature melanosomes are difficult to see in thin sections because of the orientation of the very densely packed melanosomes.

Many of the melanosomes in the zebrafish RPE cell body did not appear to be elongated, but serial section electron microscopy revealed two populations of melanosomes in the RPE; one is narrow and cylindrical, whereas the other is spherical. The greater diameter but similar volume of the spherical melanosomes suggests that they are not a precursor of the cylindrical ones, but rather are a reshaping of a similar volume carrier of melanosomal content. Previous studies have indicated that distinct melanosome morphology can arise from the production of eumelanin versus pheomelanin (Slominski et al., 2004). The black/brown eumelanin is deposited upon PMEL fibrils, leading to the formation of elliptical melanosomes (Marks and Seabra, 2001; Seiji et al., 1963). The way in which the red/blonde pheomelanin is deposited is less well characterised but might involve deposition on a vesiculoglobular matrix that results in a spherical rather than elliptical shape (Jimbow et al., 1984; Jimbow et al., 1979), somewhat like that which has been proposed to occur in fish (Turner et al., 1975). In the present study, it was not possible to distinguish between eu- and pheomelanin, but analytical electron microscopy studies have indicated that most mammalian RPE melanosomes contain a mixture of eumelanin and pheomelanin (Biesemeier et al., 2011), suggesting that the two populations, cylindrical and spherical, are not likely to simply represent eumelanosomes and pheomelanosomes, respectively.

In contrast to mammals, zebrafish express two forms of PMEL. PMELa is expressed in the RPE and melanocytes and has a longer RPT domain than PMELb and mammalian PMEL. Additionally, PMELb is exclusively expressed in the RPE (Schonthaler et al., 2005). The RPT domain regulates the packing of PMEL fibrils (Leonhardt et al., 2013). Knockdown of zebrafish PMELa causes a significant reduction in the number of cylindrical melanosomes, similar to the phenotype of PMEL inactivation in mice (Hellström et al., 2011). In addition, without a PMEL fibril matrix, a large portion of melanosomes has irregular melanin deposition and uneven melanosome morphology. A smaller effect is observed following knockdown of PMELb. These differences in the effects of knockdown of PMELa and PMELb could reflect differences in expression levels in the RPE. Alternatively, the smaller RPT and PKD regions, which together regulate amyloid formation and fibrillar packing, could imply a lesser role in fibril formation in RPE melanosome biogenesis. PMEL inactivation in mice leads to a 40–50% reduction in eumelanin content in the hair (Hellström et al., 2011), supporting a potential relationship between PMEL-dependent cylindrical melanosome shape and eumelanin deposition. The demonstration of fibrils within immature melanosomes, together with the loss of cylindrical melanosomes when knocking down PMELa, provides substantial evidence that the eumelanin model of melanosome biogenesis occurs in a similar manner in zebrafish and mammalian RPE. Conceivably, profiles suggestive of melanin deposition around the ILVs of MVBs that have previously been described in fish RPE (Turner et al., 1975) might represent a subset of melanosomes in which pheomelanin is deposited. Alternatively, the orientation of the melanosomes might not be appropriate to visualise fibrils in the plane of section, as we found that serial section analysis was necessary to visualise fibrils in zebrafish RPE melanosomes.

Although both spherical and cylindrical melanosomes were detectable in the RPE cell body only cylindrical melanosomes entered the apical processes. When the generation of cylindrical melanosomes was inhibited using PMELa MOs, movement into the apical processes was also inhibited. Taken together, these findings reveal a relationship between melanosome shape and distribution. There are a number of ways in which the round-ended cylindrical shape of the melanosome could facilitate movement into the apical processes. The high curvature at the melanosome apices, together with the narrow diameter, likely facilitates movement within the narrow diameter apical processes, the membrane of which must distend in order to accommodate the melanosome (Futter et al., 2004). Additionally, the large surface∶volume ratio of a cylinder provides a greater surface for interaction with the molecular machinery that moves the melanosome within the processes. In mammalian RPE, the Rab27a–MyRIP–myosin-VIIa complex regulates the transfer of melanosomes from the microtubule-rich cell body to the actin-rich apical processes (Futter et al., 2004; Gibbs et al., 2004; Lopes et al., 2007a). Teleosts also have bundles of uniformly oriented actin filaments in the apical processes that are tightly associated with melanosomes (Futter et al., 2004; King-Smith et al., 2014), and Rab27a has been found to be associated with fish RPE melanosomes (McNeil et al., 2004). However, mutants of the zebrafish homolog of myosin VIIa, myo7aa, do not display a defect in melanosome movement (Wasfy et al., 2014), suggesting the involvement of other motor proteins including myosin II (Barsoum and King-Smith, 2007).

The cylindrical shape of the melanosome likely has direct functional consequences, in addition to regulating its localisation. Compared with a sphere, it presents a smaller surface area to the direction of incoming light, thus maximising light access to the outer segments, while presenting a greater surface area to the lateral surfaces of the outer segments to maximise absorption of light scatter.

Knocking down PMELa not only affects melanosomes of the RPE but also affects photoreceptor outer segments, which were either missing or disrupted in thin section electron microscopy. Although we cannot exclude the possibility that reduced overall numbers of RPE melanosomes affected photoreceptor outer segments, the almost complete loss of melanosomes in the apical processes, compared with the much less affected cell body melanosomes, suggests that loss of melanosomes in the apical processes is the primary cause of the photoreceptor defects. The loss of RPE apical melanosomes and, hence, reduced protection from harmful backscattered light could explain this effect. Additionally, melanosomes within the apical processes might play a structural role in supporting the outer segments, a role that would be facilitated by the increased melanosome packing density that can be achieved by a cylindrical versus a spherical shape. In Usher syndrome (Liu et al., 1998; Liu et al., 1999) and X-linked choroideremia (MacDonald et al., 1998; Seabra, 1996), which also involve defects in melanosome movement into the apical processes due to loss of function of myosin VIIa and Rep1, respectively, the photoreceptors also gradually degenerate. However, unlike PMEL, both myosin VIIa and Rep1 are also expressed in the photoreceptors and so trafficking defects within the photoreceptors, as well as in the RPE, contribute to the observed retinal degeneration.

We have shown that key mammalian regulators of melanosome biogenesis perform similar roles in zebrafish and that the PMEL-dependent fibrils generate the cylindrical shape of melanosomes that is necessary to access the apical processes of the RPE. Given the timing of melanosome biogenesis and the extensive melanosome movement in zebrafish RPE, this will be an ideal model for the study of molecular mechanisms and functional significance of melanosome biogenesis and movement specifically in the RPE. Additionally, how organelle size, number and shape relate to their function is of increasing interest. In the zebrafish RPE, melanosome number can be altered through OA1 depletion, without changing melanosome size (at this early developmental stage), thus allowing number to regulate the size of this organellar compartment, as is usual for a multicopy organelle (Chan and Marshall, 2010). However, differential melanosome functioning can be achieved simply by PMEL-dependent shape change, which modifies localisation, without affecting the overall size of the organellar compartment. This allows for the optimal generation of a multipurpose organelle. The zebrafish model will likely allow for deliberate modulation of the critical parameters to provide a test-bed for whether and how these different populations are controlled.

## MATERIALS AND METHODS

### Zebrafish embryo injections

General maintenance, collection and staging of the zebrafish were carried out according to the Zebrafish Book (Westerfield, 1994). The approximate stages are given in days post-fertilisation (dpf) at 28°C, according to morphological criteria (Kimmel et al., 1995). Morpholinos (Gene Tools, LLC, Philomath, OR) were designed complementary to the 5′ sequence near the start of translation (atg) or at splice junctions (sp) of *pmela*, *pmelb*, *gpr143* and *tyr* sequences, summarised in supplementary material Table S1. Up to 2 ng of morpholino in 1.4 nl of Danieau's solution was microinjected into the yolk of one- to two-cell-stage embryos per experiment, with vehicle-only controls. The embryos and larvae were examined and imaged using a Leica dissecting microscope and Openlab 5.5.2.

### Electron microscopy

Zebrafish embryos (2 and 5 dpf) were fixed with 2% paraformaldehyde-2% glutaraldehyde prior to incubation with 1% osmium tetroxide-1% potassium ferrocyanide. Following dehydration in an ethanol series and propylene oxide, the zebrafish were embedded in epon resin. 70-nm sections and 200-nm serial sections were cut and examined on a Jeol 1010 TEM and imaged using a Gatan Orius SC1000B charge-coupled device camera. At least three zebrafish for each morpholino were used for quantification in each experiment. Montaged images of full-length retina were generated using low-magnification electron microscopy images in Photoshop and, subsequently, the threshold setting in ImageJ was used to remove all image information excluding melanosomes, allowing measurement of melanin area. ImageJ was used to measure the melanosome diameter, and circularity was calculated by dividing the width by length. IMOD (Kremer et al., 1996) was used to model melanosomes from the serial section electron microscopy data.

## Supplementary Material

Supplementary Material
