# Supplementary material for: Regulation of melanosome number, shape and movement in the zebrafish retinal pigment epithelium by OA1 and PMEL
Source: J Cell Sci. 2015 Apr 1;128(7):1400–7. doi: 10.1242/jcs.164400 (PMC4379728; doi:10.1242/jcs.164400)
Supplement: Supplementary Material [file supp_128_7_1400__index.html]

Regulation of melanosome number, shape and movement in the zebrafish retinal pigment epithelium by OA1 and PMEL — Supplementary Material 

# Regulation of melanosome number, shape and movement in the zebrafish retinal pigment epithelium by OA1 and PMEL

## JCS164400 Supplementary Material

**Files in this Data Supplement:**

- **Supplementary Material**
