# Supplementary material for: Regulation of melanosome number, shape and movement in the zebrafish retinal pigment epithelium by OA1 and PMEL
Source: J Cell Sci. 2015 Apr 1;128(7):1400–7. doi: 10.1242/jcs.164400 (PMC4379728; doi:10.1242/jcs.164400)
Supplement: Supplementary Material [file supp_128.7.1400_JCS164400.pdf]

Supplementary Figure 1 – Variation in zebrafish pigmentation in morpholinos at 2dpf. Light microscopy images show a reduction in pigmentation in OA1 MO-treated embryos at 2dpf, compared with control MO-treated embryos (Ctrl), but no clear difference at 5dpf. Tyrosinase (Tyr) MO treatment causes a dramatic loss of pigmentation at 2dpf. Although there is some recovery of pigmentation by 5dpf there is still clearly less pigmentation than controls. Scale - 500μm.

Supplementary Figure 2 – Similar melanosomal features observed in mouse and zebrafish RPE. Immature melanosomes from tyrosinase MO-treated 5dpf embryos give an appearance of dense spots when sectioned perpendicular to the striation direction. Mature melanosomes from control 5dpf embryos frequently appear to contain 'holes'. Images of immature and mature melanosomes from 8 day old and adult Blk6 mouse RPE respectively, that were embedded and sectioned in the same way and show the same features, are included for comparison.

Supplementary Figure 3 – Serial section EM analysis of tyrosinase morpholino-treated RPE reveals immature melanosomes that contain fibrils. (A – E) Serial section micrographs of the same immature melanosome in an RPE cell from a tyrosinase MO-treated zebrafish at 5dpf. (F) 3D rendering of the micrograph data reveals fibrils running through the melanosome. (E) Micrograph of immature melanosomes from tyrosinase MO-treated zebrafish showing melanosomes containing dense spots of melanin and a melanosome sectioned parallel to its long axis containing fibrils (arrow). Scale – 100nm.

Supplementary Figure 4 – Differential effects of PMELa and PMELb morpholinos on melanosome shape. A. Schematic structures of zebrafish PMELa and PMELb. PKD (polycystic kidney domain), TM (transmembrane) and RPT (repeat) regions were assigned using NCBI conserved domain search, TMHMM Server V 2.0 and EMBL-EBI RADAR respectively. B and C. Reduction in cylindrical melanosomes at 2dpf (B) and 5dpf (C) when depleting PMELa and PMELa&b. The dotted lines represent spherical melanosomes.

Supplementary Figure 1

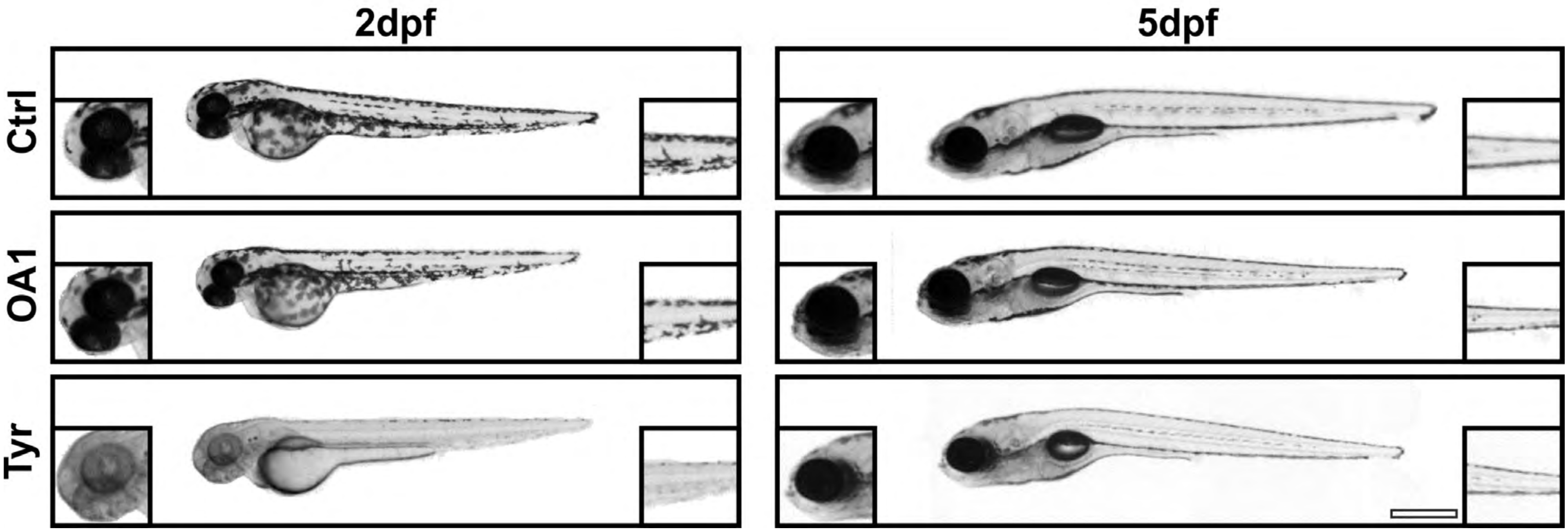

Supplementary Figure 2

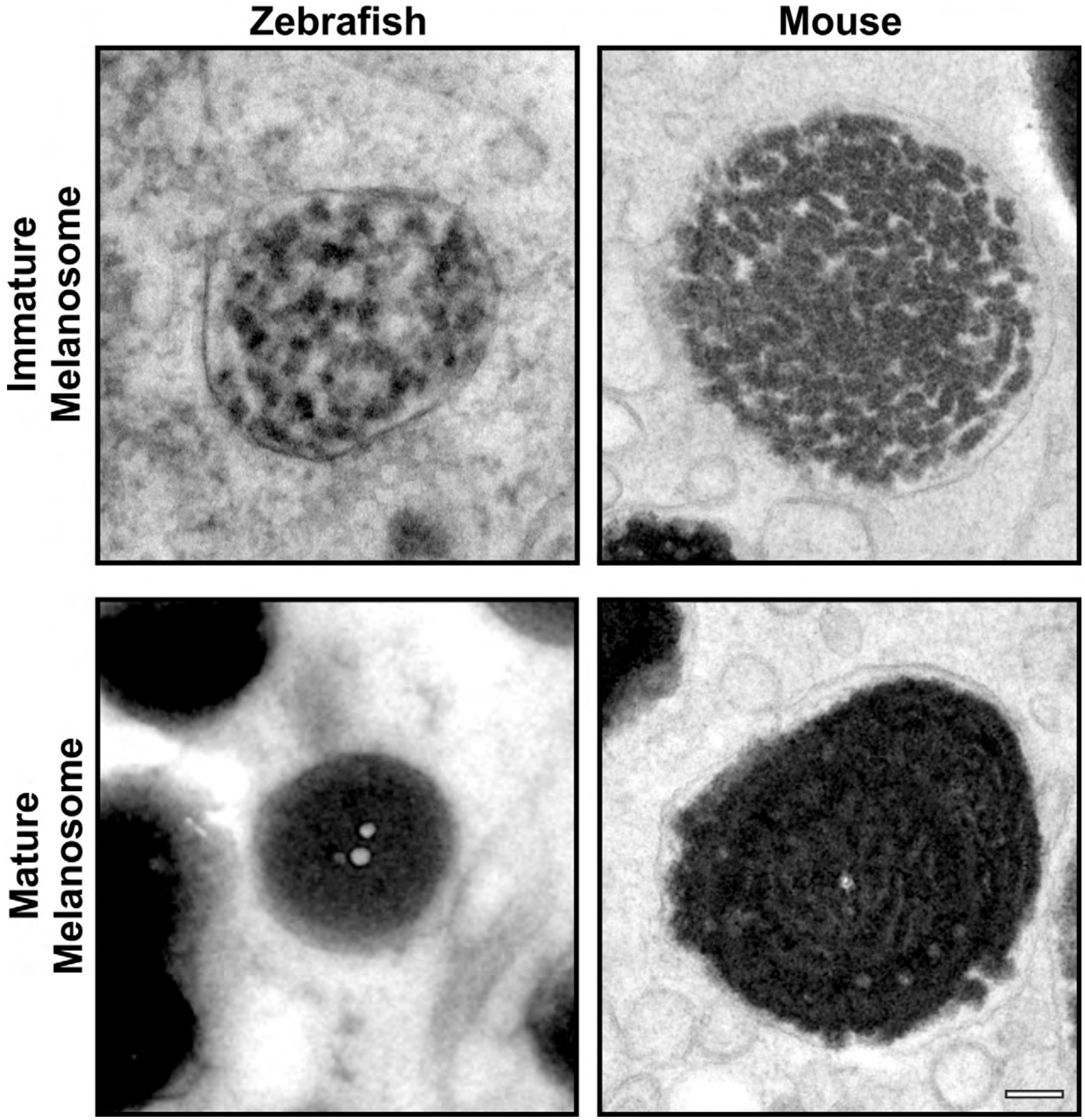

Supplementary Figure 3

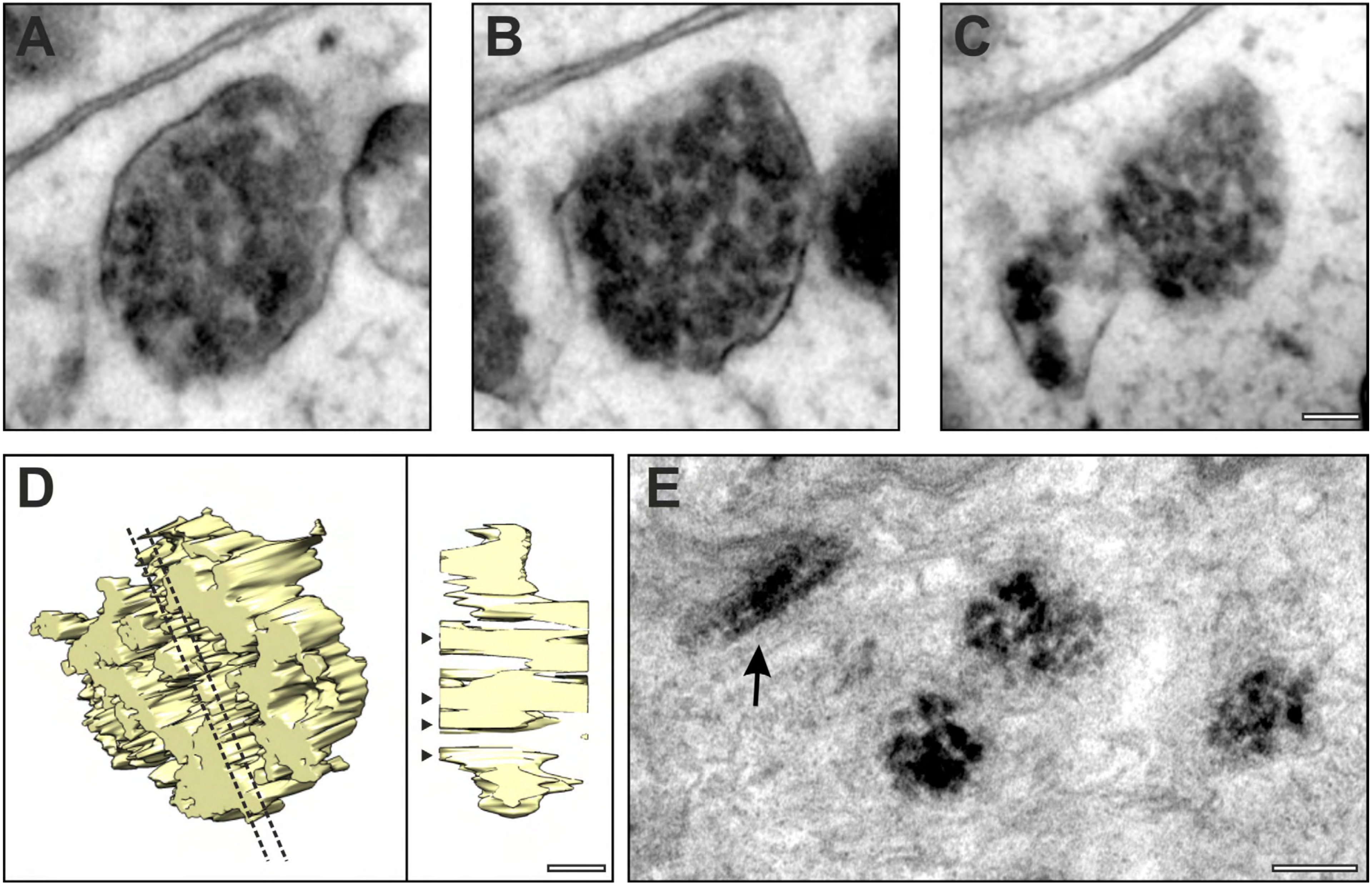

Supplementary Figure 4

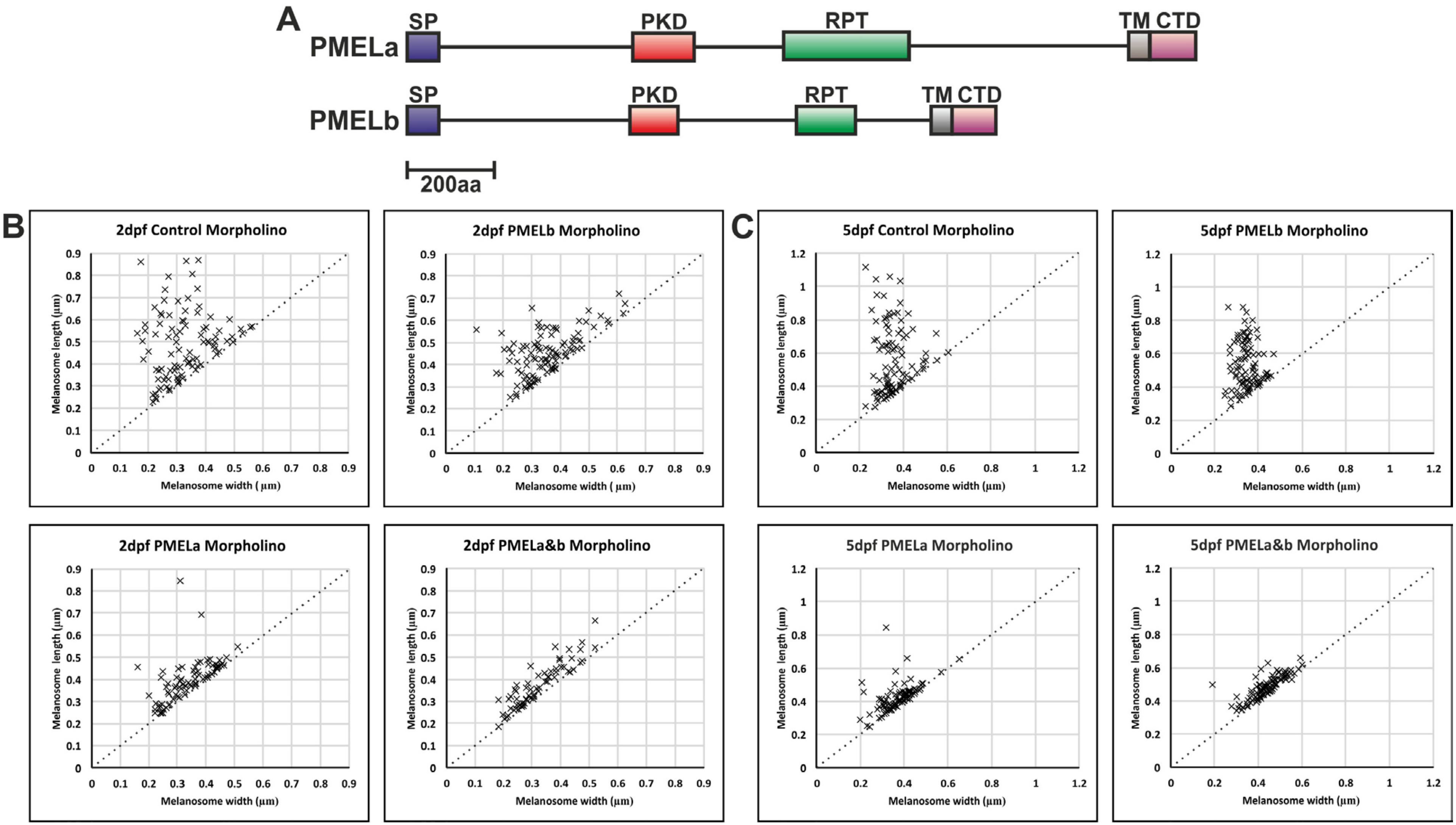

Table S1. Morpholino sequences

GENE ID	Zebrafish transcript*	MO	Sequence 5'-3'
gpr143 (OA1)	ENSDART00000047080	gpr143 atg1	AGGCCATGATGATGAAGATGAAGAA
tyr	ENSDART00000122238	tyr sp1	GAGACATGATGATGAAGAGTCGAGG
pmela	ENSDART00000123568	pmela atg1	GATGAGAGATGTCCACATGATGACC
pmelb	ENSDART00000046268	pmelb atg1	GTAGAGAATAGCTTCATTGTGTCAC

*Ensembl Identifier
